# Cost of Care for HIV-Infected Patients with Co-Occurring Substance Use Disorder or Psychiatric Disease: Report from a Large, Integrated Health Plan

**DOI:** 10.1155/2014/570546

**Published:** 2014-06-22

**Authors:** Gerald N. DeLorenze, Ai-Lin Tsai, Michael A. Horberg, Charles P. Quesenberry

**Affiliations:** ^1^Division of Research, Kaiser Permanente Northern California, 2000 Broadway, Oakland, CA 94612, USA; ^2^Mid-Atlantic Permanente Research Institute, Mid-Atlantic Permanente Medical Group, Rockville, MD 20852, USA

## Abstract

*Background*. The costs of providing care to HIV-infected (HIV+) patients with co-occurring diagnoses of substance use (SU) disorder or psychiatric disease (PD) are not well documented. It is our objective to evaluate costs in these HIV+ patients receiving care in a large health plan. *Methods*. We conducted a retrospective cohort study from 1995 to 2010 to compare costs of healthcare in HIV+ patients with and without co-occurring SU disorder and/or PD diagnoses. Estimates of proportional differences in costs (rate ratios) were obtained from repeated measures generalized linear regression. Models were stratified by cost category (e.g., inpatient, outpatient). *Results*. Mean total healthcare costs per patient per year were higher in HIV+ patients diagnosed with SU disorder or PD compared to HIV+ patients without these comorbid conditions. After controlling for confounders, total mean costs remained significantly higher in patients diagnosed with SU disorder (RR = 1.24, 95% CI = 1.18–1.31) or PD (RR = 1.19, 95% CI = 1.15–1.24). Mean outpatient care costs were significantly greater in patients with both SU disorder and PD (RR = 1.52, 95% CI = 1.41–1.64). *Conclusions*. Given these higher expenditures in the care of HIV+ patients with comorbid SU disorder and/or PD, greater efforts to facilitate SU disorder or PD treatment initiation and persistence could provide substantial savings.

## 1. Introduction

In the last 16 years the increasing efficacy and effectiveness of combination antiretroviral therapy have increased survival for HIV-infected patients [1, 2]. Thus there is increasing prevalence in the number of patients living with HIV infection [3, 4] and evidence of change in the patterns and magnitude of costs incurred in the care of HIV-infected patients [5–8]. However, less is known about the costs of care for HIV-infected patients with a dual diagnosis of substance use disorder or psychiatric disease [9, 10].

There has been increasing prevalence of substance use disorder as well as psychiatric disease diagnosed among HIV-infected patients [11–13]. In a large cohort study of HIV-infected veterans, 34% had one or more psychiatric disease diagnoses [14]. It is estimated that approximately half of HIV-infected patients have major depressive symptoms and 20%–25% meet diagnostic criteria for a depressive disorder [15]. A national survey reported that 40% of HIV-infected patients use illicit drugs [16] and >20% are diagnosed with substance use (SU) disorders, including alcohol abuse [11, 17, 18]. Evidence indicates that these more severely afflicted HIV-infected patients have greater HIV disease progression [19–21] and reduced survival when compared to patients without a SU disorder or psychiatric disease (PD) diagnosis [22–25]. Patients with SU disorders or PD diagnoses have lower quality of life compared to other HIV-infected without those comorbid conditions [26]. There is limited evidence to suggest that combination antiretroviral therapy (C-ART) may improve mental health [27]. Patients with co-occurring SU disorders or PD diagnoses are much less likely to adhere to C-ART [28–30]. Yet it is not well understood the extent to which these comorbidities affect the costs of care for HIV-infected patients. This subpopulation of HIV-infected patients with multiple conditions poses considerable challenges for adequate treatment adherence and clinical management.

In the current study we examine SU disorder and PD in relation to costs of providing care for HIV-infected patients who are members of the largest fully integrated health care plan in Northern California, where access to care is a not limiting factor. Initial analyses will determine estimated care costs for HIV-infected patients with SU disorder diagnosis or PD diagnosis. We will also examine the relative rates of health care costs in these two diagnostic groups controlling for demographic and clinical characteristics which also influence care costs.

## 2. Methods

### 2.1. Study Population

*Data Collection*. We conducted a retrospective observational cohort study from years 1995 to 2010 among HIV-infected patients who were members of the Kaiser Permanente Northern California (KPNC) health plan. KPNC is an integrated health care system with a membership of 3.5 million patients, representing 30% of the medically insured population in Northern California. HIV-infected patients are seen at medical centers throughout the KPNC 17-county catchment region. The membership is representative of the northern California population with respect to race/ethnicity, gender, and socioeconomic status, except for some underrepresentation of the lower economic spectrum [31]. For example, using data from the 2010 US Census [32], we see that the general population of northern California (counties where the KPNC members reside) is 50% non-Hispanic Caucasian; 51% of KPNC members are non-Hispanic Caucasian; 15% are Asian in both Northern California (NC) and in KPNC members; 21% of NC residents are Hispanic versus 18% Hispanic in KNPC members; 7% of NC residents are Black versus 8% of KNPC members. In NC 18% of the population had less than a high school education versus 16% in the KPNC membership; 49% of the NC population were high school graduate versus 51% in the KPNC membership; 32% of the NC population had at least one year of college versus 31% in the KPNC membership. 

The base study population consisted of 14199 HIV-infected patients who received health care at KPNC at some time between 1995 and 2010. Since 1988, the KPNC Division of Research (DOR) has maintained a surveillance system of patients who are HIV-1-seropositive, ascertained through monitoring electronic inpatient, outpatient, laboratory testing, and pharmacy dispensing databases for sentinel indicators of probable HIV-infection. HIV-1 seropositivity then is confirmed through review of patient medical records. Ascertainment of HIV-infected patients by the KPNC HIV registry has been shown to be at least 95% complete. The HIV registry contains information on patient demographics (e.g., sex, birth date, and race/ethnicity), HIV transmission risk group (men who have sex with men, injection drug use, heterosexual sex, other, and unknown), dates of known HIV infection, and AIDS diagnoses. Our study population is representative of the HIV/AIDS population residing in the western U.S [33]. In particular the KPNC HIV-infected population is similar to the California (CA), Oregon, and Nevada HIV-infected populations for gender (90% male in KPNC, 89% male in CA, 90% male in Oregon, and 88% male in Nevada), for age (54% <40 years old in KPNC, 59% in CA, 59% in Oregon, and 60% in Nevada), and for race (21% Black in KPNC, 18% in CA, 24% in Nevada, but with lesser proportion for Blacks in Oregon [7%]; 45% non-Hispanic Caucasian in KPNC, 50% in CA, 69% in Oregon, and 53% in Nevada; 20% Hispanic in KPNC, 27% in CA, 20% in Oregon, and 19% in Nevada) for years 1999–2013 [34–37]. Mortality information is obtained from hospitalization records, membership files, California death certificates, and social security administration databases. KPNC institutional review board approval with waiver of informed consent was obtained.

Patients could enter the study up until January 1, 2010, with baseline date defined as date of entry into the KPNC HIV registry, or January 1, 1995, if registry entry date was prior to 1996. Followup ended at the earliest of the following events: termination of health plan membership, death from any cause, or end of the study (December 31, 2010). Gaps in patient's health plan enrollment/membership of <3 months duration were spanned to count that time as part of continuous enrollment. Experience has shown that most KPNC patients continue to receive health care and services from KPNC during short time gaps in enrollment. Study covariates were ascertained at baseline and during followup from historical electronic administrative and clinical databases, including hospital admission/discharge/transfer data, outpatient visits, patient demographics (including race/ethnicity and HIV risk behavior), laboratory tests results, including CD4 T-cell counts and HIV-1 RNA levels, pharmacy dispensing data, radiology data, and cost of care databases.

### 2.2. Substance Use Dependence or Abuse Diagnosis

A diagnosis of ICD-9 SU dependence or abuse can be made by the patient's clinician in primary care, substance use treatment, or psychiatry as a primary or secondary diagnosis [38, 39]. Diagnostic categories include all alcoholic psychoses, drug psychoses, alcohol dependence syndrome, drug dependence (including opioid, barbiturate, sedative/tranquilizer, cocaine, cannabis, amphetamine, and hallucinogen dependence, but excluding tobacco dependence), alcohol abuse, cannabis abuse, hallucinogen abuse, barbiturate abuse, sedative/tranquilizer abuse, opioid abuse, cocaine abuse, and amphetamine abuse. Patients with one or more SU dependence or abuse diagnoses were included in this exposure group.

### 2.3. Psychiatric Disorder Diagnosis

Psychiatric diagnoses were assigned by KPNC health care providers. One or more diagnoses can be coded by ICD-9 in the KPNC administrative databases [40]. Psychiatric diagnoses selected for this study were the most common and serious psychiatric disorders diagnosed among health plan members including schizophrenic disorders (including schizoaffective type), major depressive disorder, bipolar affective disorder, neurotic disorders (including panic disorder), hysteria, phobic disorders, obsessive-compulsive disorder, anorexia nervosa, and bulimia. We examined the impact of having one or more of these psychiatric disorders in aggregate, as in prior HIV studies [41]. Within the health plan, psychiatry can be accessed directly by patients. Mild cases of depression and anxiety may be addressed in primary care with medication but moderate to severe cases are referred to psychiatry.

### 2.4. Cost of Care Data

Costs for services provided by KPNC were obtained from the Cost Management Information System (CMIS), an automated system that integrates use and financial databases. Thus, the payer perspective was adopted for the study. Costs, including program and facility overhead, are generated for services using standard accounting methods and program-specific relative value units. From these, we obtained costs of hospitalization and outpatient encounters, including emergency department and office visits as well as radiology and laboratory services. We obtained outpatient pharmacy costs from KPNC's Pharmacy Information Management System (PIMS), which records information on all prescription drugs dispensed at KPNC outpatient pharmacies. Outpatient/office visit costs are determined by the department and by human resource type (e.g., physician, and nurse practitioner). Each department/human resource type is assigned a cost by CMIS; for physicians it is the average salary in that department given the time spent for a particular visit. For services covered by KPNC but provided by non-KPNC vendors, we used payments made by KPNC to those vendors. KPNC patients do not make copayments to non-KPNC vendors. All costs were adjusted to year 2009 dollars using the Consumer Price Index.

### 2.5. Statistical Analyses

The initial analyses included descriptive statistics of mean costs per patient* per year* for total as well as inpatient outpatient and pharmacy costs. Interquartile range (25th %-ile, median, 75th %-ile) was estimated for total costs and each component category of costs. Point and interval estimates of proportional differences in costs (expressed as rate ratios) between HIV-infected patients diagnosed with and without substance use (SU) or psychiatric disorder were obtained from* generalized* linear regression models under the gamma distribution with log link (i.e., log-linear) [42]. For these log-linear gamma models, the dependent variable was the per-patient direct medical cost in each calendar quarter of followup, with an offset term of (log) months of follow-up time in each quarter to account for potential mid-quarter cohort entry and exit. In order to account for within person nonindependence in repeated measures over quarters of followup, model parameter estimation was via generalized estimating equations (GEE) assuming an autoregressive covariance structure. The primary independent variables were SU diagnosis and psychiatric diagnosis, both treated as time-dependent (yes versus no) with updating of covariates at the beginning of each quarter of followup; an HIV-infected patient was classified as having no psychiatric disease or SU disorder until date of first diagnosis. Cost ratio estimates are presented both unadjusted for covariates (crude) and with successive adjustment for an* a priori* chosen set of covariates in nested models: partial adjustment for age at time when cost occurred, calendar year when cost occurred, sex, and race/ethnicity; full adjustment with the additional covariates AIDS diagnosis (at study entry or during followup), HIV RNA levels, CD4 T-cell counts, and HIV transmission risk group. Age, CD4 T-cell counts, and HIV RNA levels were modeled as time-dependent covariates with updating at the beginning of each quarter year; laboratory variables were assessed as the most recent within the 6 months prior to first day of the quarter. For CD4 cell counts and HIV RNA levels we included missing as a covariate category in the regression models. Previous research has shown that patients with missing measurements have outcomes that are similar to patients with low CD4 counts and higher HIV RNA levels [22, 23].

We assessed total costs and costs stratified by care-setting, including outpatient pharmacy, nonpharmacy outpatient costs, inpatient costs, and other costs. Inpatient costs include the cost of inpatient stays. Other costs included skilled nursing facility stays, home health care visits, and hospice care. Nonpharmacy outpatient costs included the cost of laboratory and radiology services, emergency department visits, same day hospitalizations, clinic visits, and durable medical equipment. Because gamma distribution modeling would exclude any records with no costs, we added $1 to each care category of summarized costs in each quarter that an individual cohort member remained in the study [43, 44]. This allowed us to retain all eligible records under study (for a given quarter and care setting of cost).

In addition to point and interval estimation of SU and psychiatric disorder main effects, we also present estimates of joint effects (e.g., having both SU disorder and psychiatric disease diagnosis versus neither diagnosis) given evidence of borderline significant interaction between these two covariates and given the inherent value in directly measuring and presenting estimates of resource use in categories of joint comorbidities. These were obtained via appropriate linear combinations of parameter estimates from a fully saturated model (main effects and interaction terms), with adjustment for covariates. All data analyses were conducted using SAS software, version 9.1 (SAS, Inc, Cary, North Carolina, USA).

## 3. Results

**Figure 1:**
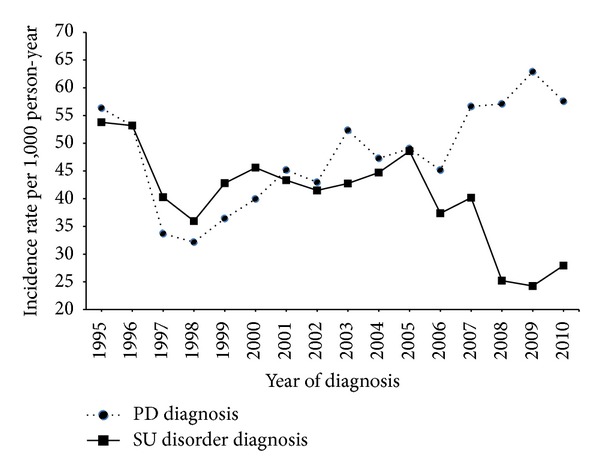
Annual incidence of substance use (SU) disorder diagnosis and psychiatric disease (PD) diagnosis in HIV-infected patients in the Kaiser Permanente Northern California health plan.

Between 1995 and 2010, there were 14199 HIV-infected patients who received care at KPNC medical facilities. The majority of patients were male (90.5%) and between the ages of 30 and 49 years old (71.1%) as presented in Table 1. Whites constituted 56% of the study sample; Hispanics were 13%; Blacks were 17.2%. Approximately 57% of patients had received an AIDS diagnosis before or during study followup. Substance use (SU) disorder was diagnosed in 23% (*n* = 3204) of patients during study followup, including 1009 patients with alcohol abuse/addiction (32%), 498 (16%) with multiple drugs abuse/addiction, 259 (9%) with amphetamine, 121 (4%) with cocaine, and 71 (2%) with opioid abuse/addiction. Of the 3530 patients who received a diagnosis of psychiatric disease (PD), 72% had major depressive disorder, 10% had panic disorder, 8% had bipolar disorder, 2% had multiple psychiatric disorders, and 2% had schizophrenia or schizoaffective disorder. The annual incidence of SU disorder and PD diagnoses has fluctuated over time in our cohort (see Figure 1). Annual incidence rates of both SU disorder and PD diagnoses declined from 1995 through 1998. Incidence trends remained similar for both types of diagnoses through 2006. After that year, incidence rates began to diverge, with PD diagnoses increasing and SU dx diagnoses decreasing through 2010.

For the entire study cohort over the duration of the follow-up period (1995–2010), total (all care provided) costs per patient* per year* averaged $30,810 (interquartile range: $9143 (25th%-ile), $16,978 (median), and $26,078 (75th%-ile)). The proportion of the total costs attributable to (1) pharmacy costs was 51.5%, (2) outpatient costs was 35.4%, and (3) inpatient care costs was 11.9%; the remaining 1.2% (other) of total costs was attributable to skilled nursing facility and hospice care costs.

**Figure 2:**
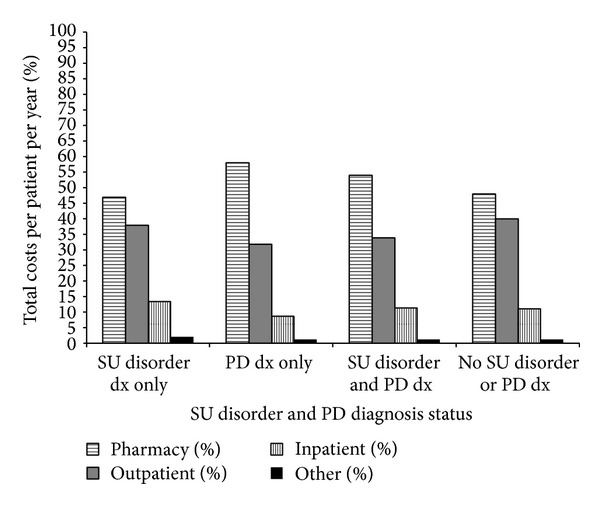
Percent of total costs per patient per year attributable to pharmacy, outpatient, inpatient, and other costs by substance use (SU) disorder and psychiatric disease (PD) diagnosis (dx) status.

In contrast, total costs per patient* per year* averaged $37333 (interquartile range: $10545, $18860, $30983) in patients diagnosed with a SU disorder only. The distribution of total costs that was attributable to pharmacy, outpatient, inpatient, and to other care (skilled nursing facility and hospice care) is presented in Figure 2. Patients diagnosed with a psychiatric disease (PD) only averaged $33037 (interquartile range: $13767, $21155, $31832) in total costs per patient* per year*. The distribution of component costs is similar to patients with SU disorder diagnosis only except that percent due to pharmacy care is higher and outpatient care is lower. For patients diagnosed with both SU disorder and PD, total costs per patient* per year* averaged $32881 (interquartile range: $14478, $22745, $34389). The distribution of the components of total costs is similar to patients diagnosed with PD only. Among patients with neither a SU disorder nor PD diagnosis, total costs per patient* per year* averaged $29142 (interquartile range: $7404, $15337, and $24243). The distribution of components of total costs is similar to patients with PD diagnosis only and to patient with both SU and PD diagnoses.

In preliminary modeling results, we found evidence of a borderline significant interaction between SU disorder diagnosis and PD diagnosis (*P* = .055 for total costs). Estimated RRs from the crude and fully adjusted GEE regression models for the joint effects of SU disorder diagnosis (yes/no) and PD diagnosis (yes/no) in relation to total cost of care and for component cost categories (e.g., inpatient costs and outpatient costs) as outcomes are presented in Table 2. The crude (unadjusted) parameter estimates (Model 1) indicate that having both a SU disorder diagnosis and a PD diagnosis is associated with significantly elevated mean total cost of care (RR = 1.53; 95% CI = 1.46 to 1.60) in comparison to patients who have neither diagnosis. A similar pattern is found for outpatient costs (RR = 1.48; 95% CI = 1.36 to 1.61) and for pharmacy costs (RR = 2.32; 95% CI = 2.24 to 2.40) in patients having both a SU disorder diagnosis and a PD diagnosis. Having a SU disorder but no PD diagnosis was associated with significantly higher mean inpatient costs (RR = 1.74; 95% CI = 1.49 to 2.02) in comparison to patients with no diagnoses; a lower (RR = 1.29) but still significant effect was observed in patients with both diagnoses in relation to mean inpatient costs. Partial adjustment by demographic characteristics (Model 2) reduced the RR estimates for having both a SU disorder diagnosis and a PD diagnosis in relation to mean total cost of care (RR = 1.35; 95% CI = 1.29 to 1.42) and to mean pharmacy cost (RR = 1.16; 95% CI = 1.11 to 1.21) outcomes, while increasing the RRs somewhat for inpatient costs. Further adjustment for clinical characteristics (model 3, fully adjusted) did not significantly alter model 2 RR estimates observed for most outcomes, with the exception that having a dual diagnosis of SU disorder and PD increased mean inpatient costs substantially (RR = 2.24; 95% CI = 1.85 to 2.70). This increase in RR is due to a very strong association between low/missing CD4 counts and per-person inpatient costs, a very strong association between high/missing HIV RNA levels and inpatient costs, and an inverse association between SU disorder/PD diagnoses and missing laboratory tests results; missing laboratory tests results data, which is very similar to low CD4 cell counts and high HIV RNA levels in terms of strength of association with inpatient costs, is more likely among those without an SU or PD diagnosis (data not shown).

## 4. Discussion

Our study presents the most recent estimates of cost for treatment and medical services provided to HIV-infected patients with comorbid diagnoses of SU disorders and/or psychiatric disease, who received care from the same fully integrated health plan. In our descriptive analyses we observed that both the mean and median total costs per patient* per year* were higher in HIV-infected patients diagnosed with SU disorder or PD compared to HIV-infected patients without these comorbid conditions. Similarly, our regression analyses found elevated mean costs associated with SU disorder and PD after adjustment for demographic and clinical covariates. In a cost of care analysis of HIV-infected veterans using a regression model approach similar to the current study, researchers observed that within a larger group of patients with SU disorders the mean total costs per patient* per year* were highest among patients diagnosed with opioid or cocaine abuse [45]. In the same study among HIV-infected veterans with PD, the highest mean total costs per patient* per year* were in patients diagnosed with schizophrenia, depression, or posttraumatic stress disorder. These higher costs occurred mostly for inpatient and outpatient care services. A study of HIV-infected patients receiving care through Medicaid in Pennsylvania during 2003 found that mean total costs per patient* per year* were substantially higher among patients with PD diagnosis compared to those with no PD diagnosis [46]. In an integration of care randomized clinical trial of HIV-infected patients who had both SU disorder and PD diagnoses in the multicenter HIV cost study cohort, researchers found that the mean annual total cost of care per patient was approximately $37932 [10] when adjusted to year 2002 dollars (Consumer Price Index), which is approximately $45235 in year 2009 dollars (adjustment made in our study); this is higher than the mean total costs of care we observed in our study for patients with both SU disorder and PD diagnoses ($32881). Reasons why KPNC costs were lower than costs estimates from other studies may be that (1) KPNC costs data are of high quality, (2) KPNC costs are lower than other providers, and (3) we have chosen to focus on provider costs. While these studies present some interesting results, their sample sizes are small (even in multicenter studies) and duration of followup is short in comparison to our study.

In our study the proportion of total costs attributable to inpatient care was slightly higher in HIV-infected patients with SU disorder compared to HIV-infected patients without a SU disorder diagnosis (13.2% versus 11.2%). A similar proportion of inpatient costs was seen in HIV-infected patients diagnosed with PD compared to HIV-infected patients with no PD diagnosis. Costs incurred during outpatient care were proportionately similar for both patients diagnosed with SU disorder and patients without a SU disorder diagnosis (37.9% versus 37.8%). The proportion of all costs incurred by pharmacy dispensing was higher in patients diagnosed with PD compared to patients without a PD diagnosis (57.9% versus 49.3%). Thus, strategies for optimizing care will be different for patients with SU disorder diagnosis, as opposed to PD diagnosis.

Examining the joint effect of having both SU disorder and PD diagnoses in relation to total costs of care showed that the significantly elevated (>50%) total costs in the crude model were reduced after adjustment for potential confounders, in comparison to patients having neither diagnosis. For inpatient costs, the RR estimates for having both diagnoses in comparison to patients without a SU disorder or PD diagnosis remained substantially greater after full adjustment of confounders and clinical characteristics including CD4 cell counts and HIV RNA levels. Schackman et al. [6] in their study of lifetime costs of HIV care in the U.S. found that the inpatient cost component of total cost of care increases from 10% in patients with CD4 cell counts >300/*μ*L to 40% in patients with ≤100/*μ*L CD4 cell counts. However, inpatient costs have not been the primary driver of total costs of care in HIV-infected patients during the C-ART era which began during 1996 [47, 48]. Pharmacy (mostly C-ART regimens in the more modern era and opportunistic infections prophylaxis) [6] and outpatient costs are the larger components of total costs of care in the C-ART era, as our data indicates.

From a review of the literature there appear to be very few studies which compare costs of providing all healthcare to the non-HIV-infected general patient populations with and without PD or SU disorder diagnoses, particularly where costs are modeled with adjustment for confounders and other cofactors [49, 50]. However, examining unadjusted costs, a study of 6500 Medicaid HMO beneficiaries observed that total costs for patients with SU disorder diagnosis were 12% higher when compared to patients with neither SU diagnosis nor PD diagnosis [51]. In the same study patients with PD diagnosis were 7% higher when compared to patients with neither PD diagnosis nor SU diagnosis. In a study conducted at KPNC among hazardous drinkers and drug users, with and without PD diagnosis, in the general patient population, a linear model (adjusted for age and sex) showed increased total costs of $188 per patient per year in comparison to patients with neither SU disorder diagnosis nor PD diagnosis [49]. This excess is much smaller than a similar comparison among the HIV-infected patients in our study.

Our study had several limitations that deserve mentioning. First, we focused exclusively on health care costs incurred within the health plan. We could not estimate costs associated with the lost productivity of HIV-infected patients (employment and income) or out-of-pocket expenses related to HIV care. Our study data were derived from health plan members in northern California and may not be similar to data from HIV-infected patients residing in other regions (e.g., the Southeast) of the U.S. Although actual costs in this health plan may not be representative of costs in other health plans or among the uninsured, the relative costs of HIV-infected patients diagnosed with SU disorder or PD compared to HIV-infected patients without those diagnoses should reflect these differences in other health care settings. There may also have been some underascertainment of HIV-infected patients with SU disorders or PD, if they did not receive a documented clinical diagnosis. However it is unlikely that patients with serious PD would not eventually come to the attention of KPNC clinicians. In addition patients with SU disorder often have trouble in their jobs, and employers will mandate SU disorder treatment (which KPNC health plan providesto most of its membership) as a requirement for continued employment. Our definition of SU disorder and PD diagnoses could be viewed as a limitation. HIV-infected patients with SU disorder and/or PD remain in those diagnostic categories from date of diagnosis to the end of followup. Some patients may recover from either or both diagnoses through appropriate treatment. However our previous research has shown that only a small proportion of patients diagnosed with SU disorder seek treatment from KPNC's chemical dependency recovery programs. While they may be seeking such care outside of KPNC, we are unable to ascertain that information.

Our results have important implications. First, as costs of caring for HIV-infected patients with PD or SU disorder are higher than patients without such diagnoses; efforts at better identifying such patients early in their HIV care are essential. Additionally, as these differences in costs vary by the comorbid diagnoses, healthcare systems should budget differently depending on their patient population (e.g., budget more for inpatient costs if the patient population has a larger HIV-infected and SU disorder diagnosed subpopulation). Interventions early in the care of these comorbid patients also may lead to greater cure or control of these comorbid conditions, leading to a cost of care profile more akin to the noncomorbid HIV-infected population.

## 5. Conclusions

In conclusion, the higher per patient expenditures (in total cost of care, inpatient costs, and outpatient costs) for the current study's HIV-infected patients diagnosed with SU disorder and PD could provide a key opportunity: if we can facilitate SU disorder or PD treatment initiation and persistence or otherwise better manage the costs of this severely burdened patient population, the potential costs savings could be substantial. As shown in other care studies from integrated care systems [52–54], better integration of HIV care and SU disorder treatment and PD treatment can reduce expenditures or cost-effectively improve health outcomes and is worth investigating. Thus far, only one investigation on the cost-effectiveness of care integration in this type of patient population has been reported [10]. Further research needs to be undertaken.

## Figures and Tables

**Table 1:** Distribution of study characteristics in HIV-infected patients receiving care at Kaiser Permanente Northern California (KPNC) health plan.

Characteristic	*N* (%)
Age (years) at entry into study	
<30	1895 (13.5%)
30–39	5492 (39.2%)
40–49	4484 (32.0%)
50–59	1704 (12.2%)
60+	439 (3.1%)
Gender	
Female	1329 (9.5%)
Male	12685 (90.5%)
Race/ethnicity	
African-American	2408 (17.2%)
Asian/Pacific Islander	612 (4.4%)
Hispanic	1831 (13.1%)
White	7806 (55.7%)
Other/unknown	1357 (9.7%)
HIV transmission risk	
Heterosexual sex	1781 (12.7%)
Men having sex with men (MSM)	9006 (64.3%)
Injection drug use and no MSM	531 (3.8%)
Injection drug use and MSM	575 (4.1%)
Coagulation disorder/transfusion	221 (1.6%)
Unknown	1900 (13.6%)
Any antiretroviral use during followup	
No	2878 (20.5)
Yes	11136 (79.5%)
AIDS diagnosis at entry or during followup	
No	5979 (42.7%)
Yes	8035 (57.3%)
CD4 counts (cells/*μ*L) at entry into study	
<100	2336 (16.7%)
100–200	1541 (11.0%)
201–349	2615 (18.7%)
350–499	2592 (18.5%)
>=500	3444 (24.6%)
Missing	1486 (10.6%)
HIV RNA level (copies/mL) at entry into study	
≥50119	2317 (16.5%)
1995–<50119	2781 (19.8%)
501–<1995	509 (3.6%)
<501	2596 (18.5%)
Missing	5811 (41.5%)
Substance use disorder/psychiatric disease diagnosis	
No SU disorder or PD diagnosis	8844 (63.1%)
SU disorder diagnosis only	1640 (11.7%)
PD diagnosis only	1966 (14.0%)
both SU disorder and PD diagnosis	1564 (11.2%)

Total	14014 (100%)

**Table 2:** Log-linear GEE regression: cost of care associated with the joint diagnoses of substance use disorder (SU) and psychiatric disease (PD), adjusted for demographic and clinical characteristics in HIV-infected patients receiving care at the KPNC health plan.

Outcome	Diagnosis	Unadjusted OR (95% C.I.)	Partially adjusted∗ OR (95% C.I.)	Fully adjusted∗∗ OR (95% C.I.)
		Model 1	Model 2	Model 3
Total costs	Both PD and SU diagnoses	1.53 (1.46, 1.60)	1.35 (1.29, 1.42)	1.35 (1.29, 1.42)
	PD diagnosis only	1.34 (1.29, 1.40)	1.21 (1.16, 1.26)	1.19 (1.15, 1.24)
	SU diagnosis only	1.35 (1.28, 1.42)	1.26 (1.20, 1.33)	1.24 (1.18, 1.31)
	Neither PD nor SU diagnosis	1.00 (ref)	1.00 (ref)	1.00 (ref)

Inpatient costs	Both PD and SU diagnoses	1.29 (1.11, 1.50)	1.67 (1.42, 1.97)	2.22 (1.85, 2.66)
	PD diagnosis only	1.03 (0.88, 1.21)	1.17 (0.98, 1.38)	1.28 (1.11, 1.49)
	SU diagnosis only	1.74 (1.49, 2.02)	1.92 (1.63, 2.27)	2.14 (1.78, 2.58)
	Neither PD nor SU diagnosis	1.00 (ref)	1.00 (ref)	1.00 (ref)

Outpatient costs	Both PD and SU diagnoses	1.48 (1.36, 1.61)	1.55 (1.43, 1.67)	1.52 (1.41, 1.64)
	PD diagnosis only	1.24 (1.16, 1.32)	1.28 (1.20, 1.36)	1.28 (1.20, 1.37)
	SU diagnosis only	1.26 (1.17, 1.37)	1.28 (1.19, 1.37)	1.27 (1.17, 1.38)
	Neither PD nor SU diagnosis	1.00 (ref)	1.00 (ref)	1.00 (ref)

Pharmacy costs	Both PD and SU diagnoses	2.32 (2.24, 2.40)	1.16 (1.11, 1.21)	1.10 (1.06, 1.14)
	PD diagnosis only	1.75 (1.70, 1.81)	1.13 (1.09, 1.17)	1.08 (1.04, 1.10)
	SU diagnosis only	1.65 (1.60, 1.71)	1.10 (1.07, 1.14)	1.06 (1.03, 1.09)
	Neither PD nor SU diagnosis	1.00 (ref)	1.00 (ref)	1.00 (ref)

*Adjusted for age at time when cost occurred, year when costs occurred, sex, and race/ethnicity.

∗∗Adjusted for age, year when costs occurred, sex, race/ethnicity, HIV transmission risk group, HIV RNA level, and CD4+ cell count (both time-dependent).

## Abbreviations

SU: Substance use
PD: Psychiatric disease
HIV: Human immunodeficiency virus
C-ART: Combination antiretroviral therapy.
